# Amyand's Hernia, State of the Art and New Points of View

**DOI:** 10.1155/2017/9598478

**Published:** 2017-09-17

**Authors:** Guido Mantovani, Michela De Angelis, Francesco Di Lecce, Annalisa Pascariello, Domenico Risio, Luigi Boccia

**Affiliations:** ^1^Division of General and Hepatobiliary Surgery, Department of Surgical Sciences, Dentistry, Gynaecology and Paediatrics, University of Verona, Verona, Italy; ^2^Department of General Surgery, Carlo Poma Hospital, Mantua, Italy

## Abstract

**Background:**

Amyand's hernia (AH) is an inguinal hernia containing the vermiform appendix, with an incidence between 0.4% and 1% of all inguinal hernias. Acute or perforated appendicitis can complicate AH.

**Case Presentation:**

A 75-year-old Caucasian man presented with incarceration of vermiform appendix in inguinal hernia sac. Diagnosis was posed preoperatively with computed tomography (CT) scan. Patient underwent urgent surgery and simultaneous appendectomy and hernia repair by Bassini's technique were performed.

**Conclusions:**

Preoperative diagnosis of AH is rare; however it could be useful for surgeon to choose operative approach. Treatment of AH depends on grade of appendix inflammation and/or perforation. The technique utilized to repair hernia depends largely on surgeon's preferences; the presence of inflamed or perforated appendix is not an absolute contraindication for using a prosthetic mesh.

## 1. Introduction

Amyand's hernia (AH) is defined as an inguinal hernia containing the vermiform appendix, whether the vermiform appendix is normal, inflamed, or perforated. The eponym comes from Claudius Amyand, an English surgeon that in 1735 performed an appendectomy, on an eleven-year-old patient, for a perforated cecal appendix incarcerated in an inguinal sac, the year after Amyand published the first paper that had ever described this type of hernia [1]. Inguinal hernia is a very common pathology, especially in men, with an estimated prevalence of 1.2% of the population [2]. Incidence of AH is estimated between 0.4% and 1% of all inguinal hernias [3]. Acute or perforated appendicitis is a possible complication of AH; inflammation of the appendix is due to its compression at the neck of the sac. Incidence of appendicitis in AH is rare, reaching about 0.1% [1], with mortality range counting from 15% to 30% in perforated appendix [4].

## 2. Presentation of Case

**Figure 1:**
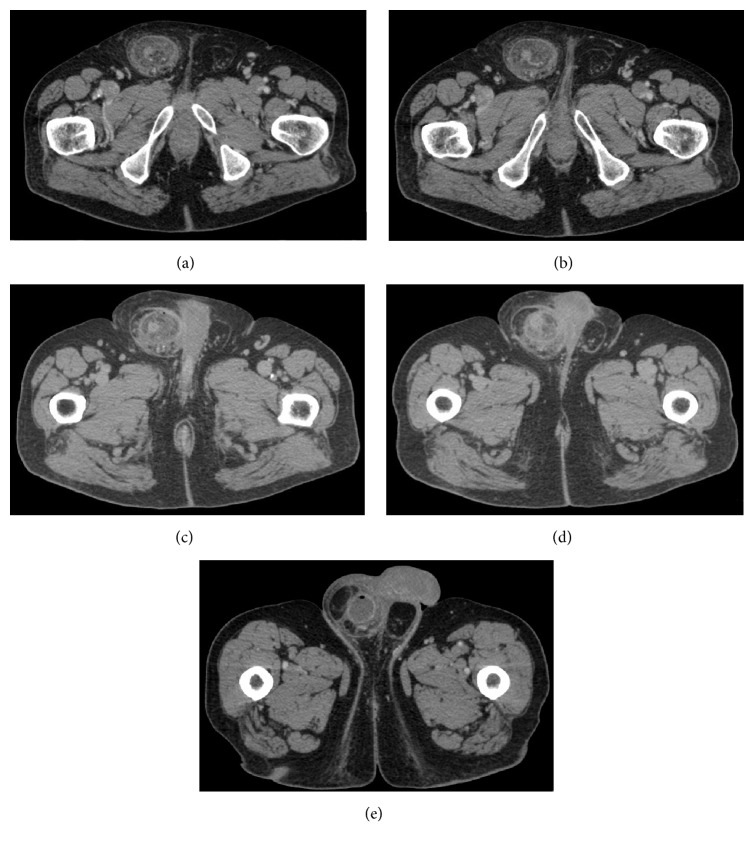
Inguinal hernia with incarceration of a part of cecum and vermiform appendix at CT scan with endovenous contrast.

Male patient, 75 years old, with history of a right inguinal mass that progressively grew over several years, went to emergency room of Mantua General Hospital for right inguinal pain, associated with fever and neutrophilic leukocytosis, without signs of intestinal obstruction. After urological assessment, with suspected diagnosis of acute epididymoorchitis, the patient was discharged with anti-inflammatory and antibiotic therapy. After 24 hours the patient came back to emergency room for fever associated with worsening of the right inguinal pain and clinical features of intestinal obstruction. At physical examination, a nonreducible right inguinal mass was noted and diagnostic suspicion of strangulated right inguinal hernia was formulated. An abdominal CT scan with endovenous contrast was performed that confirmed inguinal hernia with incarceration of a part of cecum and vermiform appendix (Figures 1(a)–1(e)). Preoperative diagnosis of Amyand's hernia was made and the patient was prepared for urgent surgery. In the operating room, an incision was made above the external inguinal ring and carried down to expose a large hernia sac which was opened, revealing the incarcerated, inflamed appendix. Appendectomy was performed, Bassini's technique was then used to repair the hernia, and drainage was posed in the right hemiscrotum. Due to the presence of an important dilatation of bowel and cecum, to better evaluate their vascularization and their tropism state, minilaparotomy was performed and pelvic drainage was placed. The patient's postoperative course was unremarkable. The pathology report described acute, phlegmonous appendicitis. Patient was discharged in fifth postoperative day. Patient follow-up at one week, one month, and one year did not reveal hernia recurrences but showed a postsurgical retraction of the spermatic cord and of the right testicle.

## 3. Discussion

Amyand's hernia is an uncommon condition characterized by the presence of the cecal appendix in the inguinal hernia sac. Amyand's hernias are classified according to the vermiform appendix characteristics: (type A) appendix without signs of inflammation, (type B) appendix with signs of inflammation, and (type C) perforated appendix [6, 16]. Preoperative diagnosis of AH is very difficult; in literature only one in 60 cases is diagnosed preoperatively [17]; AH diagnosis can be obtained by ultrasound (US) and/or computed tomography (CT).

Table 1 shows the small number of cases of AH patients in different series of literature by the years and how rarely a preoperative diagnosis was posed, particularly when CT was not performed.

US can identify an inflammatory mass in hernia sac; CT doubtlessly has more specificity and sensitivity but is usually not performed [6]. Clinical presentation changes according to AH classification. Patients with AH type A have episodic, cramping abdominal pain, rarely pyrexia, or other symptoms [18]. Patients with AH type B or C show right iliac pain, associated with vomiting, fever, and leukocytosis [19, 20]. Tenderness over McBurney's point is generally absent [21]. Differential diagnoses can be made with irreducible inguinal hernia, inguinal lymphadenitis, acute scrotum, and epididymitis. Surgeon faced by AH has to treat two of the most common surgical diseases: hernia and appendicitis. Surgeon should consider if cecal appendix in the inguinal hernia sac is inflamed, not inflamed, or perforated; after this evaluation he has to ask himself two questions: is it necessary to perform appendectomy? Is it better to repair hernia with or without prosthetic mesh?

If AH is recognized intraoperatively and a not inflamed appendix is identified in the sac, surgical treatment includes reduction of hernia and tension-free repair of the wall defect [6, 22, 23]; prophylactic appendectomy should not be performed to avoid the contamination of prosthetic mesh. In cases of AH type B or C appendectomy has to be performed; this surgery increases infection rate and possible infections of prosthesis, due to the combination of clean surgery with contaminated surgery [24]. However Chatzimavroudis in his study did not identify the presence of inflamed appendix or perforated appendix as an absolute contraindication for use a prosthetic mesh and did not describe postoperative complications after prosthetic inguinal hernia repair associated with appendectomy [25]. If the choice is not to use prosthetic mesh repair, the technique utilized to repair the defect depends largely on the surgeon preferences, but Shouldice technique is preferable because of its lower recurrence rate [26]. Nowadays, in patients with AH type B or C, we suggest to perform a prosthetic inguinal hernia repair using biological mesh to limit the risk of postoperative infection and to prevent recurrence. Laparoscopy has a marginal role in this pathology, due to the fact that diagnosis of AH is intraoperative, except for rare cases. However if preoperative diagnosis of AH is done, laparoscopic appendectomy followed by primary repair of inguinal hernia [27] or laparoscopic appendectomy and laparoscopic repair of inguinal hernia can be performed [28], with transabdominal or preperitoneal approaches [29, 30].

## 4. Conclusions

Amyand's hernia is a rare condition that surgeons have to face in their career. Preoperative diagnosis is difficult and uncommon. Treatment of AH type A is standardized, while treatment of AH types B and C is more discussed. The increasing use of biological mesh could, in the coming years, establish itself as the better technique for AH type B and C repair, limiting postoperative mesh infections. The role of laparoscopy in AH treatment is still marginal; its development strictly lies with the possibility of performing a preoperative diagnosis of AH.

## Figures and Tables

**Table 1:** AH series in the literature and their features.

Author	Year	Number of cases	Appendix feature^*∗*^	Preoperative diagnosis
Amyand [1]	1736	1	Grade C	No
Goodwin and Ghilchik [5]	1998	1	Grade B	No
Fernando and Leelaratna [6]	2002	1	Grade B	No
Breitenstein et al. [7]	2005	1	Grade B	No
Maizlin et al. [8]	2007	3	Grades A, B, C	No
Cunha et al. [9]	2009	1	Grade A	No
Yang et al. [10]	2009	4	Grade A	No
Coulier et al. [11]	2010	1	Grade B	Yes (CT)
Mai [12]	2011	1	Grade C	No
Junaid and Fawad [13]	2012	1	Grade A	No
Lombardo and Pavone [14]	2013	1	Grade A	No
Dong et al. [15]	2014	1	Grade B	Yes (CT)
Ceulemans et al. [16]	2014	1	Grade B	Yes (CT)

^*∗*^Ceulemans classification of the appendix within the hernia sac.
